# Microbiological Testing of Probiotic Preparations

**DOI:** 10.3390/ijerph19095701

**Published:** 2022-05-07

**Authors:** Anna Zawistowska-Rojek, Tomasz Zaręba, Stefan Tyski

**Affiliations:** 1 Department of Antibiotics and Microbiology, National Medicines Institute, Chełmska 30/34, 00-725 Warsaw, Poland; t.zareba@nil.gov.pl (T.Z.); s.tyski@nil.gov.pl (S.T.); 2 Department of Pharmaceutical Microbiology, Medical University of Warsaw, Banacha 1b, 02-097 Warsaw, Poland

**Keywords:** probiotic products, probiotic viability, probiotic identification, live biotherapeutic products, microbial contaminants

## Abstract

Probiotic microorganisms that are potentially beneficial to the health of the host are commercially available in a great variety of products. Not all microorganism strains present in products have proven beneficial to the health properties. These products include not only foodstuffs but also dietary supplements, food for special medical purposes, medicinal products, as well as cosmetics and medical devices. These products contain from one to a dozen bacterial strains of the same or different species and sometimes also fungal strains. Since the pro-health effects of probiotics depend on a specific strain, the number of its cells in a dose, and the lack of pathogenic microorganisms, it is extremely important to control the quality of probiotics. Depending on the classification of a given product, its form, and its content of microorganisms, the correct determination of the number of microorganisms and their identification is crucial. This article describes the culture-dependent and culture-independent methods for testing the contents of probiotic microorganisms, in addition to biochemical and genetic methods of identification. The microbiological purity requirements for various product categories are also presented. Due to numerous reports on the low quality of probiotic products available on the market, it is important to standardise research methods for this group of products and to increase the frequency of inspections of these products.

## 1. Introduction

Recently, interest in biologically active products with potentially beneficial effects on the patient or consumer has increased significantly. Some of the products containing probiotic microorganisms can be used for both therapeutic and prophylactic purposes. Depending on the indications, probiotic microorganisms are applied to humans in the form of foodstuffs, mainly fermented food, dietary supplements, foods for special medical purposes, medicinal products, or medical devices. They can also be found in cosmetics, most commonly in the forms of creams or serums [1]. In addition to products used by humans, a large group of probiotics is also used as feed supplements in animal husbandry. Probiotics belonging to the group of medicinal products are subject to clinical trials before approval by the relevant agencies and are subsequently controlled by pharmaceutical authorities to confirm the quality, effectiveness, and safety of these drugs. Furthermore, they are supervised via a system that collects data on adverse effects. Probiotics belonging to other product groups are not subject to such strict control. Numerous studies indicate the low quality of these probiotics, usually due to too low numbers of microorganisms in relation to the manufacturers’ declarations as well as the presence of microorganisms other than those declared for products dedicated for both humans [2,3,4,5,6,7] or animals [8]. Several products currently on the market contain microbes of different species, which can be a challenge during research because strains are often added to products in different amounts and have different survival rates during the storage period [7]. To distinguish medicinal products from dietary supplements or food, which, until recently, were jointly referred to as probiotics, the term “pharmabiotics” has been used in the literature for some time. It refers to biotherapeutic products containing live microorganisms, the purpose of which is to prevent or cure diseases, unlike probiotics, which are dietary supplements or food for special medical purposes and whose target group of recipients is healthy people [9].

Large and important sources of probiotics also include yoghurts, kefirs, and fermented food, most often in the form of cheeses, vegetables [10], and meats [11]. Although probiotic bacteria have been isolated from fermented foods, they can also be used to accelerate fermentation [7] and to alter the taste and texture of fermented foods [10]. In the case of meat products, the physicochemical, sensory, and functional properties may also be changed [11].

Probiotics are used not only by humans but also in animal production. In recent years, due to the limitation of the use of antibiotics, especially growth promotor factors, the use of LAB on farms to intensify meat production and prevent the development of certain pathogenic microorganisms has started [12]. Probiotics can be used as a growth stimulator for broilers [12] or in aquaculture [13]; they also influence immunity and reduce diseases and mortality in animals [12,13]. According to another study, the use of probiotics has a positive effect on the incidence of diarrhoea during piglet weaning [12].

Taking into account the large variety of probiotic products available on the market, some of which are of low microbiological quality, it is unclear how the quality of these products is controlled and whether it is possible to standardise these methods to ensure a safe product with health-promoting properties.

An extensive analysis of the available literature describing numerous different methods of determining the contents of probiotic microorganisms and their identification was performed. The aim of this review is also to present the variety of available normative methods for determining the contents, purity and identities of microorganisms in probiotic products. In addition, this review presents the advantages and disadvantages of the presented methods.

## 2. Probiotic Microorganisms

The group of probiotic organisms includes bacteria and fungi which, when administered in appropriate amounts, may exert a beneficial effect on the host’s health [14]. The most frequently used microorganisms in probiotic products are bacteria of the *Lactobacillaceae* family, in particular *L. acidophilus* and *L. rhamnosus*, as well as *L. plantarum*, *L. casei*, *L. paracasei*, and *L. salivarius* [3]. Frequently, probiotics also contain bacteria of the genera *Bifidobacterium* (*B. longum*, *B. lactis*, *B. bifidum*, *B. breve*) [15], *Lactococcus*, *Bacillus* or strains of *Streptococcus thermophiles*. Strains of yeast species, such as *Saccharomyces boulardii*, may also be present in these preparations (Table 1). *Lactobacillaceae* and *Bifidobacterium* are Gram-positive rods which produce lactic acid; they occur naturally in the digestive tracts of humans and animals. Probiotic bacteria exhibit antagonistic activity against various bacterial pathogens of the gastrointestinal tract, including *Salmonella enterica*, *Shigella sonnei*, enteropathogenic strains of *Escherichia coli* (EPEC), *Staphylococcus aureus*, *Campylobacter jejuni*, or *Clostridioides difficile*. They prevent the adhesion of these pathogens to the intestinal mucosa through competition for receptors, but they also inhibit their multiplication by competing for nutrients or producing antibacterial substances such as organic acids, hydrogen peroxide, and/or bacteriocins [16,17,18,19].

Both the FAO and the WHO [14,20] defined the criteria which should be met by strains belonging to the group of probiotic organisms. Specifically, they must not be pathogenic, i.e., they must have the GRAS (generally recognized as safe) status [21,22]. To obtain health benefits, it is necessary to apply a minimum number of 10^8^–10^11^ CFU (colony-forming units) of bacterial or yeast cells in the daily dose [23].

To assess the safety of probiotics application, the following factors should also be taken into account: a large variety of probiotic strains, the risks associated with the use of strains which do not have GRAS or QPS (qualified presumption of safety) status, as well as the possibility of interaction between the probiotic strains and the host microbiota. Probiotics may be responsible for systemic infections; excessive immune stimulation, especially in immunocompromised individuals; deleterious metabolic effects; and gene transfer [24].

Some concerns have been raised regarding strains of the genus *Enterococcus*, namely *E. durans*, *E. faecium* and *E. faecalis*, classified as probiotic bacteria (only individual strains), although they are opportunistic microorganisms capable of causing infections in humans. Numerous studies indicate the increasing importance of multidrug-resistant *Enterococcus* sp., especially those resistant to vancomycin, and the possibility of transferring resistance genes through horizontal gene transfer to other bacterial genera [25]. However, due to safety concerns and the lack of safety information and regulations, only a limited number of probiotics containing enterococci are present on the market. Moreover, these bacteria have not yet obtained the GRAS or QPS status [24,25,26,27]. Although the European Food Safety Authority (EFSA) has approved enterococci as additives in silage and food supplements [25], it does not recommend the application of enterococci in probiotic products intended for human use [27]. In Germany, the strain *E. faecalis* DSM 16431 is a compound of a drug called Symbioflor 1 and is used in acute and recurrent sinusitis and bronchitis [25,27,28]. On the other hand, the strains *E. faecium* M74 and *E. faecium* SF-68 are included in dietary supplements such as FortiFlora and Cernivet, which are considered effective and safe [25]. Enterococci are often used in probiotic products for animals due to their efficacy of action and the lack of regulations that would exclude this group of microorganisms [27].

**Table 1:** Microbial species of which individual strains are classified as probiotic.

*Lactobacillaceae*	*Bifidobacterium*	*Bacillus*	Other
*L. rhamnosus* [29,30] (Lacticaseibacillus rhamnosus *)	*B. infantis* [29,30]	*B. coagulans* [30,33]	*Saccharomyces boulardii* [29,30]
*L. acidophilus* [29,30]	*B. animalis* subsp. *lactis* [29,30]	*B. subtilis* [30,33,34,35]	*Lactococcus lactis* subsp. *lactis* [30,31]
*L. plantarum* [29,30] (Lactiplantibacillus plantarum *)	*B. bifidum* [29,30]	*B. cereus* [29,30]	*Enterococcus durans* [25,30]
*L. casei* [29,30] (Lacticaseibacillus casei *)	*B. longum* [29,30,31]	*B. clausii* [31,33]	*Enterococcus faecium* [25,30]
*L. delbrueckii subsp. bulgaricus* [29,30]	*B. breve* [29,30]	*B. licheniformis* [31,33,34]	*Enterococcus faecalis* [25]
*L. brevis* [30] (Levilactobacillus brevis *)	*B. animalis* subsp. *animalis* [32]	*B. pumilus* [34]	*Streptococcus thermophilus* [29,30,31]
*L. johnsonii* [29,30]	*B. adolescentis* [29]	*B. velezensis* [34]	*Pediococcus acidilactici* [30]
*L. fermentum* [29,30] (Limosilactobacillus fermentum *)		*B. amyloliquefaciens* [33]	*Leuconostoc mesenteroides* [30]
*L. reuteri* [29,30] (Limosilactobacillus reuteri *)			*Escherichia coli* Nissle 1917 [29,30]
*L. gasseri* [29]			
*L. paracasei* [29,30] (Lacticaseibacillus paracasei *)			
*L. salivarius* [29] (Ligilactobacillus salivarius *)			

* name according to Zheng et al., 2020 [36].

## 3. Forms of Probiotic Preparations

The probiotic preparations available on the market are present in a variety of forms. Without consideration of fermented foods such as yoghurts and kefirs as probiotics, present in almost every supermarket, the most common pharmaceutical forms of probiotics are lyophilised capsules (oral and vaginal) and oral drops. Recently, however, it has become possible to frequently encounter microencapsulated lyophilisates, which are designed to preserve the stability of probiotics during storage, protect them from harsh conditions in the upper gastrointestinal tract, release them in the colon, and facilitate probiotic microorganisms to colonise the mucosal surface [37,38,39]. The microcapsule contains a membrane surrounding a core of an extremely small diameter, ranging from a few microns to 1 mm [38,40]. The encapsulating materials are widely recognised as safe ingredients which can be used in the food industry [37]. Various materials such as alginate, xanthan gum, starch, cellulose, pectin, and chitosan are used as matrices for the microencapsulation process [38,40,41]. Alginate is the most commonly used material, due to its high membrane-forming capability, biocompatibility, and controlled release properties [41]. During the process of optimising the encapsulation of probiotics, it is extremely important to maintain the microbiological stability of the given strains as well as their functionality, safety, and effectiveness [39].

Moreover, lyophilised probiotics are also available in the form of ampoules, vials, or sachets. Probiotics in the form of tablets, as well as chocolate tablets in various forms (e.g., gummy bears), or even lollipops, are also on sale.

Probiotic microorganisms are also included in cosmetic products. The most common products of this group found on the market are creams, serums, masks, and gels, but also exfoliants, cleansers, foundations, soaps, lotions, shampoos, toothpaste, or deodorants [1,42]. Most probiotic cosmetic products do not contain live bacteria but include bacterial lysates, extracts, or products of the fermentation process, referred to as postbiotics [1,43], i.e., preparations containing non-living microorganisms and/or their components which induce a health benefit in the host [44]. Probiotic products applied to the skin surface are insufficiently controlled. There are numerous products on the market whose declared effects have not been scientifically proven [43]. The mechanism of action of probiotic cosmetics is mainly based on improving the barrier function of the epithelial layer, as well as inhibiting the growth of pathogenic microbes [1,42]. The effectiveness of this group of products has been demonstrated in the treatment of acne and atopic dermatitis [45]. Research is also carried out on the development of dressings—bandages and plasters containing probiotic bacteria (*S. salivarius* K-12, *S. salivarius* M-18 and *L. plantarum* 8P-A3), which, by producing bacteriocins, could inhibit the growth of bacteria present on the surface of the skin as well as pathogens that cause wound infections (e.g., *Cutibacterium acnes*, *S. aureus*, *Pseudomonas aeruginosa*) [46].

## 4. Determining the Count of Probiotic Microbes in Products

Determination of the microbial content in probiotic products can be performed using various methods presented in the literature. The most common ones are cultivation methods with the use of appropriate media, as well as the increasingly popular method of flow cytometry. Other methods are also described, such as fluorescence in situ hybridisation (FISH) [47] or nucleic acid abundance methods [48].

### 4.1. Cultivation Methods

Cultivation methods, such as plate count methods, are the gold standard [47]. The plate count method is simple to perform, but it requires a long incubation time and the selection of appropriate culture media. Testing the count of probiotic microbes in medicinal products, dietary supplements, or food for special medical purposes mainly depends on the composition of a given product (a preparation containing one, two, or more types of microorganisms) as well as its form (capsules, powder, drops, tablets). Numerous preparations on the market contain probiotic bacteria; however, in many of them, the number of bacteria in the product may not be consistent with the manufacturer’s declaration [2,3,4,5,6,7]. Therefore, it is important to investigate the quality of the probiotic preparations using standardised methods. In a study on the quality of probiotic preparations by Zawistowska-Rojek et al. [2], only 5 out of 25 preparations (one medicinal product, two dietary supplements, and two products classified as food for special medical purposes) contained the number of microbes above the value declared by the manufacturer in all the tested product batches. In the 10 other tested products, the number of bacteria depended on the tested product batch as well as its storage temperature, whereas in the remaining 10 products, in all tested batches, the number of microorganisms was below the manufacturer’s declaration [3]. In the studies by Mazzantini et al. [49], concerning the quality of probiotics classified as dietary supplements, 48 out of 104 analysed products did not contain all the declared species of microorganisms; 35 products had a total number of microbes lower than the declared number. However, in 22 tested products, the bacteria were not present. Medicinal products containing probiotic microorganisms were analysed in the same publication [49]; 14 out of 29 analysed drugs contained a smaller number of microorganisms than the one declared by the manufacturer. Many procedures used in research may result in discrepancies in the results presented by various authors. The method for quantifying the number of probiotic bacteria in products is described in the United States Pharmacopeia (USP) [50] and the Russian Pharmacopoeia (Ph. Ru.) [51]. According to the USP [50], the prepared sample should be dissolved in MRS broth, homogenised with a blender or stomacher, pre-incubated at room temperature, re-homogenised, and then diluted 10-fold in a peptone diluent. The method presented for determining probiotic microorganisms applies to the lactobacilli. There is a lack of information in USP concerning the methods of testing, the media used or the incubation times for products containing different types of probiotic microorganisms or several different strains of the same microbial species. In turn, according to the information given in the Russian Pharmacopoeia [51], the prepared sample should be dissolved in 0.9% NaCl and stirred 10–15 times with a pipette. After preparing a series of 10-fold dilutions in 0.9% NaCl, appropriate dilutions of the prepared suspension should be placed on Petri dishes containing an appropriate medium (Koch method), or 1 mL of the diluted suspension should be poured with a medium appropriate for the given type of bacteria (deep plate method) and incubated under appropriate conditions (Table 2) [51]. The Russian Pharmacopoeia also takes into account the instructions for quantifying bacteria of the genus *Bifidobacterium* and *E. coli* present in the same product, using Blaurock medium with sodium azide and Endo Agar [51].

In analytical tests carried out in various laboratories on the contents of probiotic microorganisms in products, described in the literature, the following procedure can be recommended. The weighed sample is dissolved in a peptone buffer [2,3,7] or phosphate buffer saline solution [5,8]. The sample is homogenised [5,7], and a series of 10-fold dilutions is prepared [2,3,6,7]. The sample should be diluted in accordance with ISO 6887-1:2000 [52]. The highest dilutions are plated on plates with a suitable medium (Table 2). After the incubation of microorganisms under appropriate conditions (Table 2), the total number of colonies on the agar plates is determined and converted to the content in the doses [6].

**Table 2:** Media and incubation conditions for individual types of probiotic microorganisms.

Microorganisms	Medium	pH	Temperature of Incubation	Conditions	Time	References
*Lactobacillaceae*	MRS Agar	5.6–5.8	37 °C ± 1 °C	5% CO_2_ or anaerobic conditions	72 h ± 3 h	[53]
	MRS Agar	6.3–6.7	38 °C ± 2 °C	anaerobic conditions	3–5 days	[50]
	LAPT Agar	6.45–6.55	37 °C ± 1 °C	anaerobic conditions	72 h ± 3 h	[54,55]
	MPC-1, MPC-2, MPC-4, MPC-5	6.2–6.6	38 °C ± 1 °C	nd	48–72 h	[51]
*Bifidobacterium* sp.	TOS-MUP	6.5–6.7	37 °C ± 1 °C	anaerobic conditions	72 h ± 3 h	[56]
	MRS Agar	6.3–6.7	38 °C ± 2 °C	anaerobic conditions	3–5 days	[50]
	Blaurock medium, MPC-5	7.0–7.4 7.0	38 °C ± 1 °C	nd	4–5 days	[51]
	RCM	nd	37 °C ± 1 °C	anaerobic conditions	72 h ± 3 h	[57]
	BSM	6.6–7.0	37 °C ± 1 °C	anaerobic conditions	24–48 h	[58]
*Streptococcus thermophilus*	M17	7.0–7.4	44 °C ± 1 °C	5% CO_2_	72 h ± 3 h	[53]
	ST Agar	6.7–6.9	37 °C	aerobic conditions	24 h	[59]
*Lactococcus* sp.	M17	7.0–7.4	20 °C ± 1 °C	aerobic conditions	72 h ± 3 h	[60]
*Bacillus* sp.	GYEA	6.6–7.0	40 °C ± 1 °C	aerobic conditions	72 h ± 3 h	[55]
	Gauze medium No.2	nd	nd	nd	nd	[51]
	MPA	7.1–7.5	nd	nd	nd	
*Saccharomyces* *boulardii*	SDA	5.4–5.8	37 °C ± 1 °C	aerobic conditions	72 h ± 3 h	[61]

nd—no data; MRS Agar—de Man, Rogosa and Sharpe Agar; TOS—TOS Propionate Agar; MUP—Lithium-Mupirocin selective supplement; BSM—Bifidobacteria selective medium; ST Agar—Streptococcus thermophilus Agar; RCM—Reinforced Clostridial Medium Agar; GYEA—Glucose Yeast Extract Agar Medium; MPA—Meat and Peptone Agar; SDA—Sabouraud Dextrose Agar.

It is extremely important to choose the right medium for the cultivation of a given type of microorganism. In the case of products containing only lactobacilli, MRS agar is the most frequently used medium [2,3,57,62]. It can also be used to test for the presence of bacteria of the genus *Bifidobacterium*. However, it is necessary to apply appropriate sterilisation conditions during the preparation of the medium. According to the information provided by the manufacturer, MRS agar should be sterilised at 121 °C for 15 min. Moreover, if the growth of the *Bifidobacterium* spp. is desired, a temperature of 118 °C for 15 min should be applied [63]. When testing a product containing both lactobacilli and *Bifidobacterium* strains, it is more practical to use two different media, e.g., MRS agar for quantifying lactobacilli and TOS-MUP for quantifying *Bifidobacterium* [60]. In such a case, the incubation conditions should be selected in such a way that the growth of *Bifidobacterium* bacteria on the MRS agar medium is excluded. The problem grows when products containing even more types of probiotic microorganisms are tested. The media should be selected in such a way that only one type of microorganism will grow on each of them. For instance, the most common medium used for the cultivation of *S. thermophilus* and *Lc. lactis* is the M17 medium. However, to obtain the growth of only the desired group of microorganisms or to determine the number of cells of each type in the product separately, it is necessary to apply different incubation conditions (Table 2) [59,60].

To mark the microorganisms of the species *L. acidophilus* in the product containing a mixture of different bacteria such as *L. delbrueckii*, *L. rhamnosus*, *L. paracasei*, *S. thermophilus*, or *Bifidobacterium*, the addition of clindamycin (0.1 mg/L) and ciprofloxacin (10.0 mg/L) to the medium is recommended. These antibiotics inhibit the growth of the mentioned species, except for *L. acidophilus* (ISO 20128:2012) [64].

In some cases, it is possible to distinguish bacterial colonies of different species remaining on the same Petri dish. The genus *Bifidobacterium* consists of strictly anaerobic bacteria which grow on the agar surface in the form of round, whitish colonies, some of them star-shaped or triple-lobed [56]. In contrast, *L. delbrueckii* subsp. *bulgaricus* forms lenticular colonies with sharply defined contours on the acidified MRS agar [53], and *L. acidophilus* grows in the form of flat, opaque grey or whitish colonies with uneven edges [64]. The species *S. thermophilus*, however, grows on this agar medium in the form of lenticular colonies [53].

Although the direct plating on Petri dishes with agar medium is the most popular method, it also has its limitations. For example, sample preparation—the rehydration of lyophilised probiotics [57]—is extremely important. Moreover, parameters such as osmolality, pH, as well as the duration and intensity of homogenisation and the ability to aggregate a given strain may significantly affect the obtained result [65].

### 4.2. Flow Cytometry

The analytical, flow cytometry method enables the qualitative and quantitative determination of microorganisms in the tested sample within a very short time, which is an advantage compared to culturing methods. The study uses fluorescent dyes, which enable the assessment of parameters related to the surface, structure, and size of cells [66]. By using fluorescence in flow cytometry, it is possible to distinguish living and dead cell populations and spores [34]. In addition, it should be emphasised that because of the use of cytometry, viable but nonculturable cells (VBNC) can be determined. These are a form of resting bacteria which can survive in unfavourable environmental conditions [67]. The VBNC cells are characterised by lack of growth on culture media but preserve cell integrity and metabolic activity [48,67,68]. The factors that may trigger the conversion of bacterial cells to the VBNC state may be, for example, inadequate acidity or osmolality of the environment, temperature changes, or a deficiency of certain nutrients [68].

In a study using flow cytometry, the number of bacterial cells was determined directly in the test sample after the addition of an appropriate dye. Bacteria capture the dye, which, under the influence of intracellular enzymes, splits and releases molecules capable of fluorescence [69]. Depending on the fluorescent dyes used, it is possible to determine the population of all cells present in the product (TO—thiazole orange) and the population of dead and damaged cells (PI—propidium iodide) [70]. Other commonly used dyes are PI/CFDA (propidium iodide/carboxyfluorescein diacetate), SYTO 24/PI (nucleic acid dye/propidium iodide), and DiOC2(3) (cyanine dye). The PI/CFDA dyes are used to mark damaged and dead cells and to determine the activity of intracellular esterase [69]. The SYTO 24/PI is another set of dyes in use. The SYTO 24 dye penetrates living and dead cells, whereas PI penetrates only those with a damaged membrane and through the cover of damaged cells; DiOC2(3) dye enables the quantification of cells containing a functioning membrane potential [69]. The three dyes presented are the standard method for quantifying lactic acid bacteria with the flow cytometry method, according to the standard ISO 19344 [71].

The results obtained with the cytometry method are expressed in units of fluorescence activity (Active Fluorescent Unit AFU/g). Additionally, it is possible to quantify the value of the non-Active Fluorescent Unit (n-AFU/g), which represents damaged and dead bacterial cells, stained with PI, as it enters cells with an intact membrane and binds to DNA. The Total Fluorescent Unit (TFU/g) represents the total number of cells as the sum of AFU and n-AFU [70].

Genovese et al. [34] used flow cytometry to determine the numbers of spores of *Bacillus subtilis*, *B. licheniformis*, *B. pumilus* and *B. velezensis* strains with the use of SYTO 24 and LDS 751 (Laser Dyes Styryl)—cell permeant nucleic acid stain. The obtained results indicate no statistically significant differences in the determination of the number of spores by flow cytometry and the use of the plating methods [34].

Comparative studies of the two methods used for the quantification of probiotic bacteria, the culture method and flow cytometry, conducted by Chiron et al. [72], demonstrated that the number of microorganisms in the analysed products was greater when flow cytometry was used for most of the analysed strains. The *B. longum* strain was an exception, for which a greater number of colony-forming units was demonstrated with classical microbiological methods. On the other hand, a study conducted by Michelutti et al. [66] showed a good correlation between both applied methods (plate culture and flow cytometry) in determining the contents of *B. animalis* and *L. acidophilus* in probiotic products. Moreover, the flow cytometry method was characterised by greater repeatability and better precision. However, the flow cytometry method is used to quantify all microbial cells in the tested sample, not only probiotic cells but also microbes contaminating the preparation, which may cause false-positive results.

### 4.3. Other Counting Methods

Other methods of microorganism counting are much less frequently used (Table 3). Fluorescence in situ hybridization (FISH) enables the enumeration of probiotic bacteria in products. The method is based on the detection of the nucleic acid nucleotide sequence with a fluorescently labelled probe that hybridises specifically to a complementary DNA sequence in an intact cell. The FISH method enables both the visualisation and quantification of bacterial strains. Moreover, it may enable the characterisation of the growth dynamics of bacteria in the environment of a given probiotic product [47]. In the studies presented by Pasulka et al. [47], the FISH method was used to determine the number of probiotic bacteria in two types of products. The first one contained the bacteria *P. acidilactici, P. pentosaceus* and *L. plantarum* as well as spores of *B. subtilis*. The number of cells estimated in the presented method was higher than the manufacturer’s declaration for all the species mentioned. The second product contained a mixture of spores of four *Bacillus* species: *B. subtilis*, *B. amyloliquefaciens*, *B. licheniformis* and *B. pumilus*. The number of estimated *Bacillus* spores was consistent with the declaration on the label [47].

Other methods, namely molecular techniques based on the detection of nucleic acid sequences, can also be used to count bacterial cells. These methods include, e.g., polymerase chain reaction (PCR), reverse transcriptase PCR (RT-PCR), or real time-quantitative polymerase chain reaction (RT-qPCR or qPCR). Quantitative polymerase chain reaction (qPCR) is a technique that enables the quantitative assessment of the microbial population using appropriate dyes and probes. Appropriate equipment is necessary, and the method facilitates the monitoring of the increase in DNA in each subsequent reaction cycle [48]. Gorsuch et al. [35] compared three methods—flow cytometry, qPCR (in which the counting is correlated with the amount of target nucleic acid), and plate count methods to count probiotic bacteria in a product that contained *P. acidilactici*, *P. pentosaceus*, *L. plantarum*, and *B. subtilis* in 20 samples of a complex probiotic product. In their study, flow cytometry and the qPCR method gave similar results, which were, however, significantly higher compared those provided by the plate method, especially in determinations performed in the later storage periods. These results suggest that some bacteria in the population entered the VBNC state and could only be counted by flow cytometry and qPCR methods [35].

Other methods, namely molecular techniques based on the detection of nucleic acid sequences, can also be used to count bacterial cells. These methods include, e.g., polymerase chain reaction (PCR), reverse transcriptase PCR (RT-PCR), or real time-quantitative polymerase chain reaction (RT-qPCR or qPCR). Quantitative polymerase chain reaction (qPCR) is a technique that enables the quantitative assessment of the microbial population using appropriate dyes and probes. Appropriate equipment is necessary, and the method facilitates the monitoring of the increase in DNA in each subsequent reaction cycle [48]. Gorsuch et al. [35] compared three methods—flow cytometry, qPCR (in which the counting is correlated with the amount of target nucleic acid), and plate count methods to count probiotic bacteria in a product that contained *P. acidilactici*, *P. pentosaceus*, *L. plantarum*, and *B. subtilis* in 20 samples of a complex probiotic product. In their study, flow cytometry and the qPCR method gave similar results, which were, however, significantly higher compared those provided by the plate method, especially in determinations performed in the later storage periods. These results suggest that some bacteria in the population entered the VBNC state and could only be counted by flow cytometry and qPCR methods [35].

## 5. Identification of Probiotic Bacteria

Identification of probiotics is usually carried out by known standard microbiological methods. According to the FAO/WHO [20] recommendations, probiotic microorganisms should have a strictly defined species classification, down to the strain level. The health effects induced by probiotic microorganisms depend on the particular strain, indicating that accurate identification is highly important. Moreover, identification down to the strain level allows for distinguishing introduced strains from those naturally occurring in a given environment [20,73]. Appropriate labelling of probiotic product packages is also extremely important. Frequently, manufacturers only provide the species name without providing details of the strain used, which may prevent the consumer from finding detailed information about the properties of a specific strain [74]. Microorganisms should be characterised with both phenotypic and genetic methods. Moreover, the FAO/WHO recommend depositing the strains in the international culture collection [20].

Initial characterisation of probiotic bacteria consists of the determination of the cells shape after staining with the Gram staining method as well as the assessment of mobility and the ability to produce catalase [53]. The easiest way to presumptively identify bacteria is through commercially available biochemical tests, e.g., API test (bioMérieux). The operating principle of these tests is the ability to assimilate, ferment, or break down specific compounds. Appropriate tests are available for various bacterial species; e.g., for lactobacilli strains and *Lactococcus*, the API 50 CHL test. Boyd et al. [75] correctly identified only 66% out of 97 tested strains of lactobacilli, using the API 50 CHL test. Identification of the bacteria belonging to the genus *Bifidobacterium* should be performed using the API 20A kit, dedicated to anaerobic bacteria. On the other hand, the identification of *S. boulardii* yeasts can be performed with the API 20C AUX test, dedicated to yeasts. In addition, the API ZYM Kit can be used to help identify bacteria and determine the potential of probiotic microorganisms [76].

The VITEK system (bioMérieux) is characterised by a similar principle of operation, where microorganisms can be identified on the basis of biochemical reactions. The VITEK 2 ANC system card allows for identifying lactobacilli to the species level and *Bifidobacterium* only to the genus level. However, the test results obtained are often inconclusive [48].

Another method of microbial identification is the BIOLOG system (Biolog Inc., Hayward, CA, USA), which is used to identify species of aerobic as well as anaerobic bacteria, yeasts, and fungi [77], including probiotic bacteria lactobacilli*, Lactococcus*, and *Bifidobacterium* [78]. This system analyses the ability of bacterial enzymes to metabolise 95 different carbon sources, making it possible to receive a “metabolic fingerprint” [78]. Moraes et al. [79] identified lactobacilli using different methods: API 50 CHL tests, the BIOLOG system, and molecular methods (16S rDNA sequencing), yielding varying results. The BIOLOG system identification yielded five strains: *E. faecalis*, four *E. durans*, two *Streptococcus* spp., and one *Lc. lactis*; twelve isolates were classified as other species, whereas five were not identified at all. Analysing the same samples using API 50 CHL tests resulted in fourteen *L. plantarum*, six *L. paracasei*, six *Lc. lactis*, and one *Lactobacillus* spp.; one sample was classified as a different species, and one was not identified at all. The authors of this publication confirmed their results using molecular identification—16S rDNA sequencing, in which 20 results were obtained, identifying the tested microorganisms as *Enterococcus* spp., five *L. plantarum*, three *Lc. lactis*, and one *Streptococcus* spp. [79].

Databases for the analysis of phenotypic results often do not take into account the latest taxonomic changes or newly described species, and therefore, the interpretation of the obtained results is not accurate.

Another method enabling accurate and prompt identification of the tested microorganism is the MALDI-TOF MS—Matrix—Assisted Laser Desorption/Ionisation—Time of Flight—Mass Spectrometry technique. This method allows a comprehensive analysis of the protein panel of a given microorganism. The test consists of analysing the spectral distribution of proteins directly in bacterial cells [78,80]. Proteins of 2–20 kDa are detected, which are both ribosomal and housekeeping proteins [81]. The obtained protein profile is compared with the data from reference spectra, on the basis of which a given microorganism can be assigned to the species level [81]. Lorbeg et al. [7] identified bacteria derived from dietary supplements using the MALDI-TOF method and subsequently confirmed the obtained results using appropriate polymerase chain reaction (PCR). Using the MALDI-TOF method, they were able to correctly identify the following species: *B. animalis*, *B. breve*, *B. longum*, *B. bifidum*, *B. infantis*, *E. faecium*, *L. acidophilus*, *L. casei*, *L. gasseri*, *L. paracasei*, *L. plantarum*, *L. reuteri*, *L. rhamnosus*, *L. salivarius*, *Lc. lactis*, *S. thermophilus*, and *S. cerevisiae*. The accuracy of this identification was also confirmed with the PCR method. Nevertheless, when the MALDI-TOF method is used, errors also occur in the identification of lactic acid bacteria, especially in the case of closely related species such as *L. casei* and *L. paracasei* [7,78,82]. Comparative studies using MALDI-TOF and PCR methods showed discrepancies in the identification of the above species [82].

The problem with unequivocal identification of a species using phenotypic methods is related to the common phenomenon of phenotypic variability, resulting, among other things, from changes in gene expression under the influence of environmental conditions. Molecular biology methods, based on the analysis of the genetic material of bacteria, are much more accurate, sensitive, and reproducible. These methods are less reliant on the growth conditions of the bacteria, allowing the microorganism to be identified not only down to the species level but even to the strain. Many different molecular biology methods are used to identify and differentiate probiotic microorganisms. The most commonly used molecular assays for the identification of lactic acid bacteria are nucleic acid amplification tests. The PCR-based research is characterised by high sensitivity and specificity. The process of identifying lactic acid bacteria is performed based on gene sequences which encode ribosomal RNA (16S, 23S, 5S), amplification of ITSs (intergenic spacer regions) located between the genes encoding the 16S and 23S rRNA (ITS-PCR) and amplification of regions between genes encoding tRNA (tDNA PCR), sequence analysis of the genes encoding 16S rRNA, 23S rRNA, or ITS, restriction analysis of the rDNA gene amplification product ARDRA (Amplified Ribosomal DNA Restriction Analysis), ribotyping and DGGE/TGGE (Denaturing Gradient Gel Electrophoresis/Temperature Gradient Gel Electrophoresis) [78,80]. In multiplex PCR, it is possible to identify several different species of probiotic bacteria in one reaction, e.g., within the family *Lactobacillaceae: L. acidophilus*, *L. delbrueckii*, *L. casei*, *L. gasseri*, *L. plantarum*, *L. reuteri*, and *L. rhamnosus* [83]. However, this method has some limitations when it comes to the identification of closely related bacteria, e.g., from the *L. acidophilus* group (*L. gallinarium* and *L. helveticus*). In this case, it is impossible to distinguish particular species [84]. Kim et al. [85] determined 37 strains of lactobacilli with the use of primers specific for the given species *L. acidophilus*, *L. plantarum* and *L. casei*. The obtained results of the analysis of 17 probiotic products showed that not all products contain bacterial species corresponding with the information provided on the package.

Another technique used for probiotics identification based on DNA amplification is RAPD (Random Amplified Polymorphic DNA). This method is based on amplification with the use of a short primer (usually about 10 nucleotides), where the ratio of G-C to A-T pairs is taken into account. The primer bonds with numerous homologous sequences in the analysed chromosomal DNA of a given species [83,86]. The method is easy to implement, cheap and can be a quick method for the simultaneous analysis of various strains of a given species [78]. However, it has a low repeatability, especially in interlaboratory conditions [83,86]. Using this method, the species *L. helveticus*, *L. sake*, *L. plantarum* and *L. delbrueckii* subsp. *bulgaricus* [78] can be successfully distinguished. Huang and Lee [87], with the use of appropriate primers, also distinguished species belonging to the *L. casei* group: *L. rhamnosus*, *L. paracasei* subsp. *tolerans*, and *L. zeae*.

Among the methods that use restrictive analysis to identify probiotic bacteria, the RFLP (Restriction Fragment Length Polymorphism) method may be employed. In this method, the differences in the band patterns reflect changes in the DNA sequence, which result in the absence or an additional restriction locus recognised by the restriction enzyme used [88]. Blaiotta et al. [89] identified lactobacilli strains by digesting the obtained amplification products with Alu I and Tac I restriction enzymes, enabling them to identify and distinguish even closely related species such as *L. acidophilus* and *L. crispatus*; *L. casei* and *L. rhamnosus*, as well as *L. acidophilus*, *L. helveticus*, and *L. amylovorus*. Moreover, with the additional use of Sau3AI or Mse I restrictase, they were able to distinguish *L. plantarum* and *L. pentosus* species.

The T-RFLP technique (Terminal Restriction Fragment Length Polymorphism) is a modification of the PCR-RFLP technique, in which the 5′-end primer is labelled with a fluorescent dye (e.g., fluorescein amidite) so that only the labelled terminal restriction fragments are detected. This method does not require conducting a culture to identify a species from a mixed bacterial population; moreover, its accuracy can be increased by using more restriction enzymes [78]. This method was used, among others, to study the intestinal microbiota [90] as well as for the identification of probiotic lactobacilli strains in intestinal samples [81,91].

Another molecular biology method used to identify probiotic strains is the AFLP (Amplified Fragment Length Polymorphism) method, based on the analysis of the entire bacterial genome. This technique employs the phenomenon of the ligation of nucleotide adapters and the selective amplification of restriction fragments. In the AFLP technique, the following restriction enzymes are used: frequently cutting (e.g., Mse I or Taq I) and rarely cutting (e.g., EcoR I or Pst I), leaving sticky ends [80,86,88]. The advantages of the AFLP method include good reproducibility and sensitivity; no sequence knowledge is required. However, the complicated procedure, with a large number of steps, an expensive process, and the need to have specialised equipment, limit this method [80,86]. However, this method is successfully used to type bacteria of the lactobacilli. Dimitrov et al. [92] typed bacteria from 49 stool samples; using the AFLP technique, 41 profiles were distinguished, whereas when using PFGE, they obtained 34 profiles, and with the use of RAPD, only 27 profiles were obtained. On the other hand, Giraffa and Neviani [93] successfully classified strains belonging to closely related species: *L. plantarum*, *L. pentosus* and *L. pseudoplantarum*. Jarocki et al. [86] found that this method has the highest potential for differentiating strains of the *L*. *casei* group.

The method in the differentiation and relationship searching of strains, recommended by the FAO/WHO, is Pulsed Field Gel Electrophoresis (PFGE) [20]. This method is also used to test for probiotic bacteria. It is based on the digestion of chromosomal DNA with rarely cutting restriction enzymes, e.g., ApaI, AscI, SmaI, XbaI [94,95], followed by separation of the obtained digestion products in agarose gel in an alternating electric field [89]. This method has a very high differentiating potential and a very high reproducibility, but it is also extremely laborious and time-consuming. Desai et al. [96], using the PFGE method, typed strains closely related to the *L. casei* group: *L. casei*, *L. paracasei*, *L. rhamnosus*, and *L. zeae*. Xu et al. [94] digested the chromosomal DNA of 33 lactobacilli strains with AscI restrictase, obtaining 17 different pulsed-field profiles belonging to the following species: *L. rhamnosus, L. paracasei*, *L. plantarum*, *L. acidophilus*, *L. fermentum, L. curvatus*, and *L. delbrueckii* subsp. *lactis*.

The gold standard method that serves both to identify and to determine the LAB drug resistance profile is the whole genome sequencing method (WGS). The identification of strains using WGS can be performed using one of the available methods—single nucleotide polymorphism (SNP) analysis or the gene-by gene analysis method. The SNP method consists in comparing the genome of a given bacterium with a reference genome, as a result of which information about nucleotide differences is obtained. In turn, the gene-based method can be used to analyse the genetic relationship between LAB strains [80]. Special tools such as Mauve or Mummer are used for WGS analysis. Thanks to the possibility of comparing the genomes of two strains contained in the database, it is possible to distinguish strains that differ even by a single nucleotide, this makes it possible to conclude that the two strains are different, even if no phenotypic differences are identified [65]. The described method of identification, despite its accuracy, is not common in microbiological laboratories due to high costs. It is also worth adding that in order to be able to commercially use the identification of LABs derived from probiotic products by the WGS method, it will be necessary for manufacturers to include the nucleotide sequences of the strains used in appropriate and publicly available databases [65].

## 6. Microbiological Purity of Probiotic Medicinal Products and Dietary Supplements

Probiotic products, both those classified as medicinal products and food, should meet several quality requirements which are regulated depending on the status of the product. Regardless of the classification of the product as food or medicine, the product should not be contaminated with pathogenic bacteria, such as *E. coli* or *Salmonella* sp. Depending on the consumer groups for these products, consideration should also be given to excluding the presence of other pathogens such as *S. aureus*, *P. aeruginosa*, *Listeria monocytogenes*, *Clostridioides* or *Cronobacter sakazakii* in infant products. The requirements to be met by individual product groups are regulated by the European Pharmacopoeia (Ph. Eur.) [97,98,99], the United States Pharmacopeia (USP) [50,100,101,102], the Food and Drug Administration (FDA) [103] and the European Commission [104].

According to the monographs of the European Pharmacopoeia [97,98,99], in products containing live microorganisms (Live Biotherapeutic Products, LBP), depending on the route of administration of a given preparation, different maximum aerobic microbial contamination counts (AMCC) and total yeast and mould contamination counts (YMCC) should be estimated (Table 4). To determine these contaminations of LBP in the presence of probiotic strains (lactic acid bacteria, *Bacillus clausii* spores, yeast *S. cerevisiae* var. *boulardii*), various media and incubation conditions should be used, tailored to the specifics of the test product and the presence of the microorganisms in it (Table 4). Moreover, depending on the administration route, the presence of certain pathogenic microorganisms (*E. coli* in oral preparations and *P. aeruginosa*, *S. aureus* and *Candida albicans* in vaginal preparations) should be excluded (Table 4).

The US Pharmacopeia specifies the microbial purity requirements for products classified as both medicinal products and dietary supplements (Table 4 and Table 5). Depending on the microorganisms contained in the oral product (non-spore-forming bacteria, e.g., lactobacilli and *Bifidobacterium*, spore-forming bacteria, yeasts and moulds), there are maximum permissible counts of contaminating microorganisms [50,100]. In addition, undesirable microorganisms such as *E. coli* or *Salmonella* sp. are specified. If there is a risk of contaminating raw materials or the finished product, the presence of *L. monocytogenes*, *S. aureus* and *P. aeruginosa* should also be excluded, whereas in the products intended for infants, bacteria such as *Clostridium perfringens* and *Cronobacter sakazakii* must also be excluded [50,101].

Documents published by the FDA and the European Commission (Table 5) regarding food exclude the presence of the microorganisms in products, such as *Salmonella* [103,104], *Cronobacter* spp. [103], *E. coli* [103], *Enterobacteriaceae* [103], *Enterobacter sakazakii* [104], *L. monocytogenes* [104].

The FDA [105], like the standard ISO 17516:2014 [106], also specifies the requirements for the microbiological purity of cosmetics (Table 5). The requirements for this product group concern the total number of mesophilic aerobic microorganisms, both bacteria, yeasts and moulds, which should not exceed 1 × 10^3^ CFU in 1 mL or 1 g of the product; preparations used in the vicinity of the eyes are an exception [105,106]. When applied to mucous membranes [106] and to children under 3 years of age [106], for whom the given limits are lower in amount, in the case of FDA requirements, to ≤5 × 10^2^ CFU per 1 g [102], and in the case of ISO requirements ≤ 1 × 10^2^ CFU per 1 g or 1 mL [106]. Additionally, the presence of certain microorganisms in cosmetic products should be excluded, e.g., *S. aureus* [105,106], *P. aeruginosa* [105,106], *Streptococcus pyogenes* [105], *Klebsiella pneumoniae* [105], *E. coli* [106] or *C. albicans* [106] (Table 5).

Mazzantini et al. [49] collected results on the purity of probiotic products from various countries. In the presented studies, the most common microorganism that contaminated the products was *E. faecium*, even at the level of 10^9^ CFU/dose. In addition, contamination with microorganisms such as *Acinetobacter baumannii* (10^11^ CFU/dose), *Lysinibacillus fusiformis* (10^11^ CFU/dose), *B. cereus* (10^10^ CFU/dose), *Bacillus leantus* (10^9^ CFU/dose), and *Staphylococcus* spp. (10^2^ CFU/dose) was detected [49].

**Table 5:** Acceptance criteria of dietary supplements [50,100,101,102], food for special medical purposes [103,104] and cosmetics [105,106].

DIETARY SUPPLEMENTS, FOOD FOR SPECIAL MEDICAL PURPOSES			
Documents	TAMC	TYMC	Specified Microorganisms
FDA [103]	5 × 10^2^	nd	Absence of *Cronobacter* spp. per 10 g Absence of *Salmonella* per 25 g Absence of *E. coli* per 1 g Absence of *Enterobacteriaceae* per 10 g
USP [50,100,101,102]	5 × 10^3^	10^2^	Absence of *E. coli* per 10 g Absence of *Salmonella* per 10 g Absence of *L. monocytogenes*, *S. aureus*, *P. aeruginosa* if there is a risk of contamination of the product Absence of *C. perfringens* and *C. sakazakii* in infant products
Commission Regulation (EC) No 1441/2007 [104] Regulation on microbiological criteria for foodstuffs	nd	nd	Absence of *L. monocytogenes* per 25 g Absence of *Salmonella* per 25 g Absence of *Enterobacteriaceae* per 10 g
**COSMETICS**			
**Documents**	**Total Number of Mesophilic Aerobic Microorganisms (Bacteria Plus Yeasts and Moulds)**		**Specified Microorganisms**
FDA [105]	≤5 × 10^2^ CFU per 1g—cosmetics applied around the eyes ≤1 × 10^3^ CFU per 1g—other cosmetic products		Absence of: *S. aureus*, *S. pyogenes*, *P. aeruginosa K. pneumoniae*
ISO 17516 [106]	≤1 × 10^2^ CFU per 1 g or 1 mL—cosmetic products intended for children under three years of age, applied around the eyes or on the mucous membranes ≤1 × 10^3^ CFU per 1 g or 1 mL—other cosmetic product		Absence of *E. coli* per 1 g or 1 mL Absence of *S. aureus* per 1 g or 1 mL Absence of *P. aeruginosa* per 1 g or 1 mL Absence of *C. albicans* per 1 g or 1 mL

nd—no data.

## 7. Conclusions

An increasing number of probiotic products appear on the market. In the literature, there is a large amount of information about the incorrect number of probiotic microorganisms, contamination of the tested products at a very high level, and the lack of proper labelling of the strains included in the composition of the preparations.

There is no doubt that methods of testing the contents of probiotic products, especially the proper preparation of the sample and the selection of the appropriate method for counting and identification of microorganisms, are necessary. However, there are no detailed, universal guidelines for testing these products, especially when they contain many different types of microorganisms (strains of the same or different species and genera), which may cause differences in the results obtained by laboratories. The different survival times of microorganisms in the product also affect the identification of the strains declared by the manufacturers, which often, during the shelf life of the product, are found in very low numbers that are not sufficient to provide the health benefits to the host in any way, which is the basic task of probiotics.

Besides, the probiotics contaminants may include pathogenic microorganisms, which suggests that products containing live microorganisms, regardless of whether they belong to the category of medicinal products, dietary supplements, food or cosmetics, may not be safe and should be subject to strict quality control.

Taking into account reports on the poor survival of microorganisms in products, they should be also subjected to stability tests, similarly to medicinal products, to eliminate poor-quality preparations or to shorten the validity period. Detailed information about the strains contained in particular product should be provided by the manufacturers on the product package or in informational materials. Not only the generic or species name but also strain designation should be stated, because the properties of probiotics are strain-dependent.

Based on this review, a substantial amount of work needs to be done to ensure that the probiotic products available on the market are of good quality, safe and fulfil a pro-health function.

## Figures and Tables

**Table 3:** Determining the count of probiotic microbes—culture independent methods.

Methods	References
Flow cytometry	[67,68,69,70,71]
FISH (Fluorescence in situ hybridization)	[47]
PCR methods (PCR, RT-PCR, RT-qPCR, qPCR)	[35,48]

**Table 4:** Acceptance criteria for microbiological quality and cultivation conditions for medicinal products containing probiotic microorganisms.

European Pharmacopoeia [97,98,99]					
Route of Administration	AMCC *		YMCC*		Specified Microorganisms
	Acceptance Criteria	Medium and Incubation Conditions	Acceptance Criteria	Medium and Incubation Conditions	
Non-aqueous preparations for oral use	10^3^ CFU/g or CFU/mL	LBP * containing lactic acid bacteria: - Sugar-free agar plates (30–35 °C, 72 h) - Casein soya bean digest agar plates supplemented with 5% of sheep blood (30–35 °C, 44–48 h) LBP containing *Bacillus clausii* spores—sporulating agar (33–37 °C, 48 h) LBP containing *Saccharomyces cerevisiae* var*. boulardii*—casein soya bean digest agar containing cycloheximide (30–35 °C, 3–5 days)	10^2^ CFU/g or CFU/mL	LBP containing bacteria—Sabouraud -dextrose agar with chloramphenicol—(20–25 °C, 5–7 days) LBP containing *Saccharomyces* var. *boulardii*—Sabouraud-dextrose agar supplemented with chloramphenicol and cycloheximide, Czapek-Dox agar, potato dextrose agar (20–25 °C, 5–7 days)	Absence of *E. coli* per 1 g or 1 mL
Aqueous preparations for oral use	10^2^ CFU/g or CFU/mL		10^1^ CFU/g or CFU/mL		Absence of *E. coli* per 1 g or 1 mL
Vaginal use	10^2^ CFU/g or CFU/mL		10^1^ CFU/g or CFU/mL		Absence of: *P. aeruginosa*, *S. aureus*, *C. albicans* per 1 g or 1 mL
**United States Pharmacopeia** [50,100,101,102]					
**Probiotic products for oral use**	TAMC *		TYMC *		**Specified microorganisms**
Non-spore-forming bacteria	<5 × 10^3^ CFU/g (except lactic acid bacteria)		<10^2^ CFU/g		Absence of *E. coli* per 10 g Absence of *Salmonella* per 10 g Absence of *L. monocytogenes, S. aureus*, *P. aeruginosa* if there is a risk of contamination of the product Absence of *C. perfringens* and *C. sakazakii* in infant products
Spore-forming bacteria	Not applicable		<10^2^ CFU/g		
Yeasts and moulds	<1 × 10^3^ CFU/g		Not applicable		

* AMCC—aerobic microbial contamination count, YMCC—yeast and moulds contamination count, TAMC—total aerobic microbial count, TYMC—total yeast and mould count, LBP—live botherapeutic products.

## Data Availability

Not applicable.
